# Hypoxia Pre-Conditioned Embryonic Mesenchymal Stem Cell Secretome Reduces IL-10 Production by Peripheral Blood Mononuclear Cells

**DOI:** 10.6091/.21.1.24

**Published:** 2017-01

**Authors:** Majid Lotfinia, Shirin Lak, Nastaran Mohammadi Ghahhari, Behrooz Johari, Faezeh Maghsood, Sara Parsania, Bahareh Sadegh Tabrizi, Mehdi Kadivar

**Affiliations:** Department of Biochemistry, Pasteur Institute of Iran, Tehran, Iran

**Keywords:** Embryonic stem cell, Mesenchymal stem cell, Hypoxia

## Abstract

**Background:**

Mesenchymal stem cells (MSCs) are important candidates for MSC-based cellular therapy. Current paradigm states that MSCs support local progenitor cells in damaged tissue through paracrine signaling. Therefore, the study of paracrine effects and secretome of MSCs could lead to the appreciation of mechanisms and molecules associated with the therapeutic effects of these cells. This study analyzed anti-inflammatory and immune-modulatory effects of MSC secretomes derived from embryonic stem cells (ESCs) and bone marrow cells after hypoxia and normoxia preconditioning.

**Methods:**

ESCs differentiated into MSCs and characterized by flow cytometry as well as by differentiation into adipocytes and osteoblasts. The experimental groups were consisted of individual groups of ESC-MSCs and BM-MSCs (bone marrow-derived mesenchymal stromal cells), which were preconditioned with either hypoxia or normoxia for 24, 48 and 72 h. After collecting the cell-free medium from each treatment, secretomes were concentrated by centrifugal filters. Using a peripheral blood mononuclear cell (PBMC) assay and ELISA, IL-10 concentration in PBMCs was evaluated after their incubation with different secretomes from preconditioned and non-preconditioned MSCs.

**Results:**

A significant difference was observed between ESC-MSC normoxia and ESC-MSC hypoxia in IL-10 concentration, and normoxia secretomes increased IL-10 secretion from PBMCs. Moreover, the strongest IL-10 secretion from PBMCs could be detected after the stimulation by ESC-MSC conditioned secretomes, but not BM-MSC conditioned medium.

**Conclusions:**

Human hypoxia preconditioned ESC-MSC secretome indicated stronger immune-modulatory effects compared to BM-MSC conditioned medium. It could be suggested that induced MSCs confer less immune-modulatory effects but produce more inflammatory molecules such as tumor necrosis factor α, which needs further investigation.

## INTRODUCTION

Mesenchymal stem cells (MSCs) are important candidates in cellular therapy for treatment of various diseases[1]. Studies have demonstrated that after transplantation, MSCs can repair damaged tissue through paracrine effects[^2^,^3^]. Cell-free culture supernatant (secretome) of MSCs mediates therapeutic effect of these cells; therefore, secretome therapy may be replaced by conventional MSC therapies. For cellular therapy, MSCs are generally isolated from bone marrow. However, bone marrow-derived mesenchymal stromal cells (BM-MSCs) have limitations, such as difficulties in their isolation from the tissue, limited long-term proliferative capacity and the small number of isolated cells. Embryonic stem cells (ESCs) have been indicated to have the ability of differentiating into ESC-MSCs[4,5]. An unlimited number of MSCs can be produced from pluripotent stem cells. Besides, compared to BM-MSCs, ESC-MSCs have stronger inhibitory effects on cells of the immune system[6-9]. One study has demonstrated that the addition of BM-MSC secretome to peripheral blood mononuclear cells (PBMCs) increased IL-10 secretion[10]. In addition, some studies have suggested that MSC culture supernatant obtained after hypoxic preconditioning improves therapeutic effect of these cells compared to the normoxic culture[11-13].

Until now, several clinical trials have used MSCs to treat autoimmune diseases, such as diabetes, rheumatoid arthritis, Crohn’s disease, lupus erythematosus and multiple sclerosis, and some of these studies have found improvements in patient’s condition[1,14-20]. The secretome of MSCs are composed of two groups of factors. Trophic factors induce the regeneration of damaged tissue through the inhibition of apoptosis, stimulation of mitosis, differentiation of tissue-intrinsic stem cells and promotion of angiogenesis. The second group consists of immune-modulatory factors that control the immune system responses and reduce inflammation in damaged tissue[21]. To modify the immune system, MSCs suppress the proliferation and activation of T-, B-, natural killer and dendritic cells and stimulate regulatory T-cells[22]. MSC secretomes have been revealed to have several growth factors, cytokines, extracellular matrix proteins and microRNA-containing exosomes[23]. Currently, studies have concentrated on identifying the function of MSCs in the regulation of immune responses. Therefore, to understand the mechanisms and molecules associated with therapeutic effects of MSCs, detailed studies and evaluation of the paracrine effects of MSCs secretome are required.

## MATERIALS AND METHODS

### Cultivation of human embryonic stem cells

**Fig. 1:**
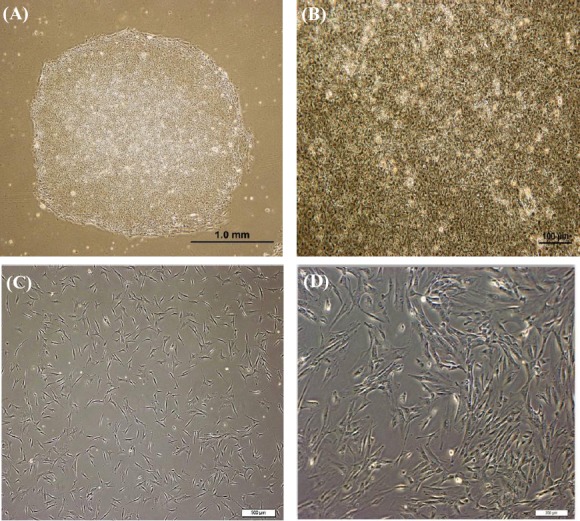
Mesenchymal stem cells (MSCs) differentiated from embryonic stem cells. A) A colony of embryonic stem cells (ESC) cells at passage 8 on the matrigel. Differentiated cells did not exist in the center and around the colonies, showing the ideal optimization of cell culture conditions; B) ESC colonies from close (10×); C) The morphology of fibroblast-like mesenchymal stem cells derived from ESCs at passage 3; D) Phase-contrast image of ESC-MSCs (20×).

Human ESCs (hESCs) at passage 8 were purchased from the Royan Institute (Tehran, Iran) and cultured feeder-free on 60-mm matrigel (0.34 mg/ml)-coated plates (Sigma Aldrich, Germany) and incubated at 37°C, 5% CO_2_, and 95% humidity[24]. hESC culture medium consisted of DMEM/F12 (Gibco, Germany), 20% knockout serum (Gibco, Germany), 2 mM L-glutamine (Gibco, Germany), 1% non-essential amino acids (NEAA) (Gibco, Germany), 100 U/mL penicillin (Gibco, Germany), 100 μg/mL streptomycin (Gibco, Germany), 0.1 mM β-mercaptoethanol (Sigma Aldrich, Germany), 100 ng/ml fibroblast growth factor (FGF) (Sigma Aldrich, Germany), and 1% Insulin-Transferrin-Selenium (Gibco, Germany). Medium was changed every day, and the cells were passaged weekly. Cells were first washed with PBS. Subsequently, differentiated surrounding and central parts of the colonies were separated with a sterile sampler tip. Colonies were cut and exposed to 1 mL DMEM/F12, including an equal volume of collagenase type IV (1 mg/ml) and dispase (2 mg/ml) at 37°C for 5-10 min. Colonies were then washed with PBS and collected for culturing on the matrigel plates (Fig. 1).

### Differentiation of human embryonic stem cells into mesenchymal stem cells

To differentiate hESCs into MSCs, Hwang *et al*.[25] protocol was used. For the formation of embryoid bodies, hESCs were cultured in non-adherent culture dish (Greiner bio-one, GmbH, Austria) without bFGF for 10 days. Embryoid bodies were grown in D-10 culture medium (DMEM, including 10% FBS and 2 mM L-glutamine) and plated on 0.1% gelatin (Sigma Aldrich, Germany)-coated dishes. Cells were then trypsinized and cultured at a high density in the same plates. Next, 95% confluent hESCs were passaged at a ratio of 1:3. After about 3 passages, a homogenous population of the cells was obtained with a fibroblast-like morphology.

### Characterization of cells

When fibroblast-like cells at passage 5 reached to 80% confluency, supernatant was removed, and cells were washed with PBS. Cells were trypsinized by 2 ml of 0.05% trypsin and counted. After centrifugation and washing, the cell pellet was dissolved in PBS containing 2% FBS and kept at 37°C for 20 min. Next, 2 μl isotype control and mesenchymal marker antibodies were added to 1.5 ml vials containing 200,000 cells/100 ml and kept at 4°C for 30 min. After the samples were washed with PBS, fluorescent labeling was tested using FACSCalibur flow cytometer (BD) and analyzed by the Flowing software (ver. 2.4)[26,27].

### Osteogenic and adipogenic differentiation of mesenchymal stem cells

For osteogenic differentiation, cells were cultured in D10 culture medium with 200 μM ascorbic acid, 10 mM β-glycerophosphate and 0.1 μM dexamethasone at 37°C, 5% CO_2_ and 20% O_2_ for 21 days. After fixation with methanol, cells were stained with Alizarin Red[28,29]. For adipogenic differentiation, cells were exposed to DMEM/F12 medium containing 20% knockout serum, 2 mM L-glutamine, 1% NEAA, 100 U/mL penicillin, 100 μg/mL streptomycin, 0.1 mM β-mercaptoethanol (Sigma Aldrich, Germany), 100 ng/ml FGF, and 1% Insulin-Transferrin-Selenium, 5% CO_2_ and 5% O_2_ (hypoxia) at 37°C for 21 days. After fixation with paraformaldehyde, cells were stained with Oil Red O[30].

### Preparation of mesenchymal stem cell secretome

At about 90% confluency, cells were incubated with DMEM without serum, including 2 mM L-glutamine and 0.1% human serum albumin in hypoxia (1% O_2_) and normoxia (20% O_2_) at 37°C and 5% CO_2_. After 24, 48 and 72 hours, cell-free supernatants were collected and centrifuged at 1500 ×g for 15 minutes to remove the cell debris. For ultracentrifugation, 30 kDa molecular mass cut-off ultrafiltration membranes (Millipore, Billerica, MA, USA) were used, and the samples were centrifuged at 5000 ×g for 1.5 h. Secretomes were transferred to tubes and kept at -20°C[12].

### Hypoxia conditioning

Hypoxic conditions were created by incubation of MSCs in a modular incubator chamber (Billups-Rothenberg, Del Mar, CA, USA) at 37°C for 24, 48 and 72 h and flushed with a gas mixture consisting of 1% O2, 5% CO_2_, and 94% N_2_. To perform hypoxia test, cells in passage 12 were used[13,31].

### Isolation of peripheral blood mononuclear cell

PBMCs were isolated from 10 ml fresh blood samples using Ficoll gradient (density=1.077 g/cc; GE Healthcare, Uppsala, Sweden). PBMCs (10^5^) were used per well of 96-well plates with an equal volume of 50 μl from different secretomes and C10 culture medium (RPMI, 10% FBS, 1% L-glutamine, 1% NEAA, 100 U/mL penicillin, 100μg/mL streptomycin, 0.1 mM β-mercaptoethanol (Sigma Aldrich, Germany). After incubating with the conditioned medium for 18 hours, PBMCs cells were stimulated with 30 μg/ml lipopolysaccharide for 5 hours, and IL-10 secretion into the supernatant was measured via ELISA.

### Determination of IL-10 production

The ELISA kit by Thermo Scientific was used according to the manufacturers’ instructions. Production of IL-10 by PBMCs was determined by ELISA, and absorbance was measured at 450 nm using a microplate reader. Results were then compared with a standard curve plotted absorbance (Y axis) against the concentration (X axis).

### Statistical analysis

ELISA results were analyzed by the unpaired student’s t-test (GraphPadInStat version 3). Data were represented as the mean of the samples±standard deviation. *P*<0.05 values were considered statistically significant.

## RESULTS

### Cell characterization

**Fig. 2:**
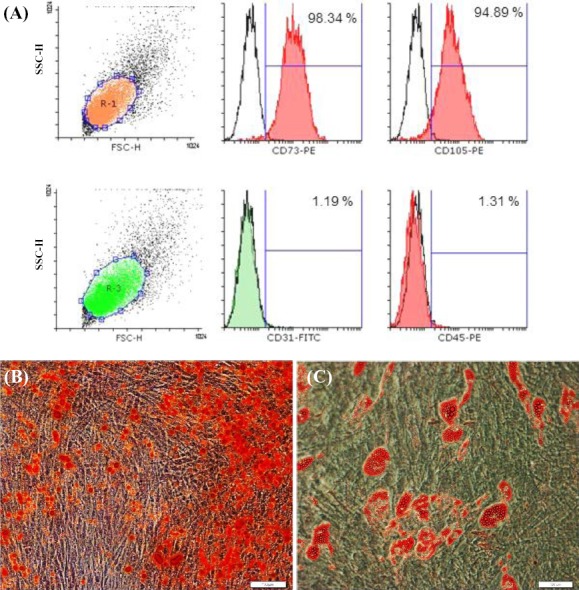
Characterizations and differentiation of embryonic stem cells-mesenchymal stem cells (ESC-MSCs). A) Flow cytometry analysis for mesenchymal markers CD73 and CD105, hematopoietic marker CD45 and endothelial marker CD31, showing the expression of mesenchymal markers on the cells; B) Osteogenic differentiation of ESC-MSCs and staining with Alizarin Red (20×). Red points show calcium deposits in the extracellular matrix;. C) Adipogenic differentiation of ESC-MSCs and Oil Red O staining, showing that cells were positively stained for red fat granules in the cytoplasm. FSC, forward scatter; SSC-H, side scatter histogram

Immuno-phenotyping of the ESC-MSCs by flow cytometry showed that these cells express mesenchymal markers such as CD73 and CD105 and poorly express hematopoietic (CD45) and endothelial (CD31) markers (Fig. 2A). Furthermore, MSCs were capable of differentiating into adipogenic and osteogenic lineages (Figs.2B and 2C).

### IL-10 production of peripheral blood mononuclear cells in hypoxic and normoxic conditions

The objective of this study was first to differentiate ESCs into ESC-MSCs. MSCs were characterized using flow cytometry and *in vitro* differentiation into adipocytes and osteoblasts. Characterized MSCs were cultured under hypoxic and normoxic conditions and after collecting the conditioned medium from each treatment, secretomes were processed for a higher concentration by ultracentrifugation. Finally, ELISA assay was used to detect the effects of different secretomes from hypoxic and normoxic culture conditions on IL-10 secretion from PBMCs. The results showed that ESC-MSCs expressed mesenchymal markers and had the potential to differentiate into bone and fat tissue lineages. When preconditioned with ESC-MSC secretomes, PBMCs produced higher levels of anti-inflammatory IL-10 compared to non-treated cells. Secretomes obtained from normoxia-preconditioned ESC-MSCs increased IL-10 secretion compared to hypoxia-preconditioned secretomes. Since increased IL-10 secretion from PBMCs is an indicator of immune-modulation, the present study indicated that hypoxic preconditioning could decrease the immune-modulatory effects of MSCs on the immune system.

**Fig. 3:**
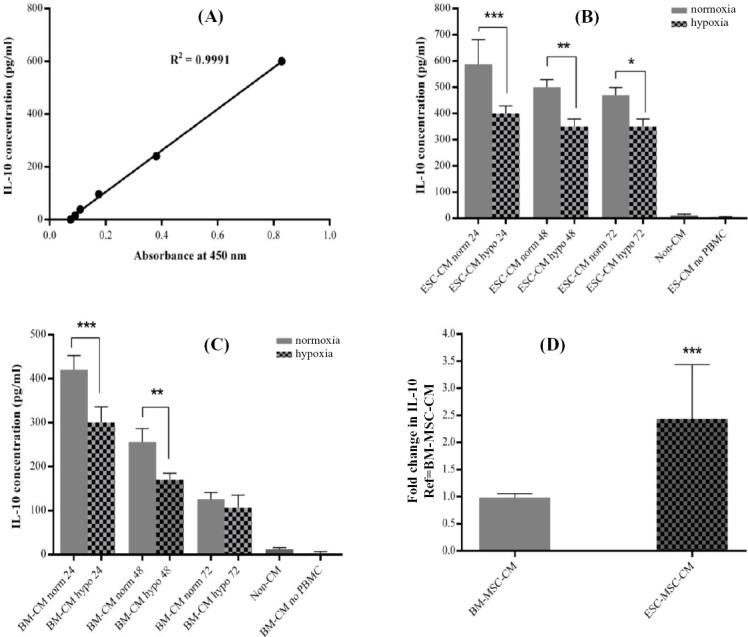
The immune-modulatory properties of mesenchymal stem cells (MSCs) derived from embryonic stem cells (ESCs) and blood mononuclear cells (BMCs) in an *in vitro* model. A) The ELISA standard curve was prepared by making serial dilutions of standard with known concentration, from the standard stock solution. All experiments were performed in triplicate. Error bars represent the standard deviation (SD), and each column represents the mean±SD of three independent experiments. The optical density of the standard samples of recombinant IL-10 protein was recorded at a wavelength of 450 λ. The standard curve was created by plotting the average absorbance obtained for each standard concentration on the vertical (Y) axis vs. the corresponding IL-10 concentration (pg/mL) on the horizontal (X) axis; B and C) PBMCs response to different hypoxic and normoxic exposure times in different experiments. IL-10 level exhibited a time-dependent decrease in response to either hypoxia or normoxia. MSC secretomes collected in either hypoxia or normoxia increased IL-10 secretion form PBMCs, compared to non-CM cells. MSC-conditioned medium (CM) normoxia at 24 h showed stronger effect to increase IL-10 secretion compared to MSC-CM hypoxia (***P<0.001). Same results were observed for 48-h group (**P<0.01); D) ESC-MSC CM increased IL-10 secretion from PBMCs compared to BM-MSC CM. no means without.

BM-MSCs have been widely described to have anti-inflammatory properties; however, no study have reported the immune-modulatory effects of ESC-MSCs. Using a recently developed potency assay, this study examined IL-10 secretion *in vitro* by human PBMCs to analyze immune-modulatory activities of pre-conditioned hESC-MSC-secretomes. The results showed that pre-conditioned MSC secretomes increased IL-10 secretion from PBMCs. Moreover, hypoxia secretomes largely reduced IL-10 secretion compared to those collected under normoxia. It is important to consider that IL-10 was not present in MSC-secretomes, and other secreted factors may result in the induction of IL-10 secretion from PBMCs. The strongest IL-10 secretion from PBMCs was observed after the stimulation by ESC-MSCs secretomes. It is worth to mention that BM-MSCs secretomes did not change IL-10 secretion from PBMCs (Fig. 3).

## DISCUSSION

In the present study, a simple assay was developed to evaluate the factors secreted by MSCs. The method uses a human PBMC inflammation assay to measure the anti-inflammatory activity of MSC secretome in conditioned medium. Using ELISA, this technique assesses the production of IL-10 as an immune-modulatory factor secreted by MSCs. PBMCs contain a variety of immune system cells such as T-, B- and natural killer and immature dendritic cells. IL-10 is an anti-inflammatory cytokine suppressing auto-immune injuries. Lymphocytes and monocytes generally secrete this cytokine; however, other PBMCs can synthesize and release IL-10[32]. Recent *in vivo* studies have observed an increased level of IL-10 after transplanting MSCs or injecting the cell-free medium of these cells to animal models[33-35]. Although cell-cell interactions between MSCs and different cells of the immune system play a significant role in immune-modulation, the secretion of soluble factors is more important to suppress the immune system[6]. In 2014, Milwid *et al*.[36] used the same inflammation assay and compared cell-secreted proteins from treated cells with an enhanced level of IL-10 and discovered two immune-modulatory proteins, microfibrillar-associated protein 5 and proenkephalin in the secretome of BM-MSCs. In another study, Yang *et al*.[37] have indicated that the co- cultivation of MSCs and splenocytes increases IL-10 secretion from splenocytes. However, when they used 1-methyl-D-tryptophan inhibitor against indoleamine 2,3-dioxygenase (IDO), the paracrine effects of MSCs on IL-10 secretion was reduced, which suggests IDO as a critical immune-modulatory factor in MSC secretome. Moreover, simultaneous cultivation of dendritic cells and MSCs reduced the expression of major histocompatibility complex II, CD11c, CD83 and IL-2 but increased IL-10 secretion from these cells. It has been shown that MSCs prevent the activation and proliferation of natural killer cells in the presence of IL-15 and IL-2 by releasing effective molecules such as prostaglandin E2, IDO and sHLA-G5[1]. Therefore, T lymphocytes, natural killer cells, and dendritic cells can be isolated from PBMCs to be individually tested for the effects of MSC secretome on these different immune system cells. MSCs derived from pluripotent stem cells can also inhibit the proliferation of T-cell and dendritic cell maturation, indicating their strong immune-modulatory capacity to suppress the innate and acquired immune system responses[38].

The present study demonstrated that preconditioned hypoxia and normoxia-preconditioned MSC secretomes could increase IL-10 secretion from PBMCs. Furthermore, stimulation with preconditioned secretomes from both hESC-MSCs and BM-MSCs increases IL-10 secretion from PBMCs. However, the strongest IL-10 secretion from PBMCs could be detected after stimulation by hESC-MSCs secretomes. On the other hand, hypoxia secretomes reduced IL-10 secretion from PBMCs more than those collected under normoxia. Other studies reported that the trophic effect of preconditioned MSCs increased in hypoxi[12,13,31]. In 2014, Chen and colleagues[39] revealed that BM-MSC secretomes, collected after hypoxia conditioning, could considerably augment keratinocyte and fibroblast proliferation. However, normal O_2_ concentration had minor chemo-attractive and mitogenic effects on these cells.

Immune-modulatory effects of MSCs under hypoxia conditions have been less studied. Our results suggest that hypoxia induces MSCs to produce less immune-modulatory factors, but more inflammatory molecules. Hypoxia may increase the expression level of inflammatory cytokines such as tumor necrosis α and IL-1β in MSCs[40]. Therefore, the reduction of the MSCs immune-modulatory effects may cause by an increase in the expression of inflammatory cytokines. Proteomics analysis of hESC-MSC secretome helps to identify unknown factors that modulate the immune responses. Hypoxia-inducible factor (HIF) is the main regulator of the hypoxic condition, and when MSCs are exposed to hypoxia, this factor increases stemness features of cells and reduces cellular aging[41]. Nevertheless, the relationship between immune-modulatory properties of MSCs and activation of HIF remains unknown, but in hypoxia, several cells in the immune system improve their features by increasing the expression of HIF[42]. In conclusion, similar studies using *in vivo* models would help to clarify the function of MSC secretome under different hypoxia and normoxia conditions.
